# The Southern Dental Association

**Published:** 1870-04

**Authors:** 


### THE SOUTHERN DENTAL ASSOCIATION.

It was our privileged good fortune to attend the meeting of the Southern Dental Association, held in New Orleans.

This being our first trip to the far South, we might write much about what we saw by the way, and in that goodly city. But we have seen all this so much better written than we could hope to do, that we forbear.

With many of the profession, whom we met there, we had a previous acquaintance, but we formed many new acquaintances, and all of the most agreeable character; and what we saw more than verified the enlarged ideas we had entertained of their large hearted and warm hospitality, both professionally and socially.

The Association continued its session four days. There were present, in all, about seventy members of the profession. A number of excellent papers were read, and the subjects presented in them were discussed with a readiness and thoroughness we have rarely seen excelled. Like all new organizations, more time was, doubtless, expended in mere business matters, and parliamentary routine, than will be after a few more meetings; there was, however, less of this than we have frequently seen.

The profession of New Orleans gave to the Association a splendid entertainment, which was enjoyed to the full by all present.

We have no where seen the profession in a state of more entire harmony and co-operation than in New Orleans. There is amongst its members, no feeling of jealousy or hostility. There seemed to be prevalent with every one a warm desire for, and a hearty co-operation in the accomplishment of that which shall result in the greatest good to the whole.

It will be a glorious time when our profession, in every city and section of the country, shall have that harmony and unity of purpose that exists with our brethren in New Orleans. T.

